# Advancing environmental interfacial chemistry of fine particles: from classical models to multi-factor coupling framework

**DOI:** 10.1093/nsr/nwaf175

**Published:** 2025-04-28

**Authors:** Jincai Zhao, Baoshan Xing

**Affiliations:** Key Laboratory of Photochemistry, CAS Research/Education Center for Excellence in Molecular Sciences, Institute of Chemistry, Chinese Academy of Sciences, China; Stockbridge School of Agriculture, University of Massachusetts, USA

Environmental fine particles are micro- and nanoscale solid particles (typically below several micrometers), representing a metastable state between dissolved substances and macroscopic particles, and they are ubiquitous in the atmosphere, water, and soil [1–3]. Due to their high specific surface area and surface energy, fine particles exhibit remarkable chemical reactivity, and their interfacial behaviors and processes (e.g. adsorption and dissolution) play a pivotal role in the migration, transformation, and stabilization of pollutants, particularly heavy metals [4,5]. However, classical models face significant limitations in analyzing these intricate interfacial behaviors and processes, making it challenging to fully reveal the function and behavior of fine particles in complex environments. Therefore, elucidating the mechanisms behind the interfacial behaviors of fine particles has become a pressing scientific challenge in the field of environmental chemistry.

To address this challenge, Lin *et al*. developed a multi-factor coupling theoretical framework for interfacial activity of fine particles, which quantitatively reveals the synergistic regulatory effects of particle size and specific surface energy on interfacial behaviors, thereby advancing the understanding of the interfacial chemistry of environmental fine particles [6]. Specifically, by extending the Langmuir adsorption model to incorporate the effects of particle size and surface defects on adsorption, they proposed the Extended Interfacial Activity Equation (1), ${Q}_{\mathrm{B}} = \frac{{Z\delta b{x}_{\mathrm{B}}( {{\mathrm{aq}}} )}}{{1 + \delta b{x}_{\mathrm{B}}( {{\mathrm{aq}}} )}}\cdot\frac{1}{r}$. They further extended the Gibbs–Thomson equation by incorporating adsorption-induced regulation of specific surface energy, leading to the derivation of the Extended Interfacial Activity Equation (2), ${\mathrm{ln}}{x}_{\mathrm{A}}( {{\mathrm{aq}}} ) = \frac{{\gamma + {\mathrm{\sigma }}}}{{1 + \delta b{x}_{\mathrm{B}}( {{\mathrm{aq}}} )}}\cdot\frac{{\mathrm{L}}}{r} + C$. This theoretical framework illustrates the complexity of fine particle interfacial behaviors under the coupling of multiple factors. For example, increasing the specific surface energy or decreasing particle size enhances dissolution, while adsorption suppresses dissolution by reducing the effective specific surface energy. This ‘adsorption-dissolution’ coupling mechanism reveals the behavioral patterns of fine particles in complex environments and provides theoretical support to explain the experimentally observed inhibitory effect of adsorption on dissolution [7].

In previous studies, Lin *et al*. have validated the critical role of the adsorption and dissolution effects of fine

particles in heavy metal pollution control [4,5,8]. For example, the ‘re-yellowing’ phenomenon of reduced chromium ore processing residue (rCOPR), in which the concentration of Cr(VI) rebounds over time, has been a significant problem plaguing the industry for more than 30 years [9]. They discovered experimentally that the small-size effect of fine particles in rCOPR induced accelerated dissolution, rendering conventional stabilization technologies ineffective [5,8]. By applying the proposed theoretical framework, they quantitatively elucidated the synergistic regulatory effects of particle size and specific surface energy on the migration and transformation of heavy metals, providing a scientific basis for addressing this long-standing challenge.

**Figure 1.**
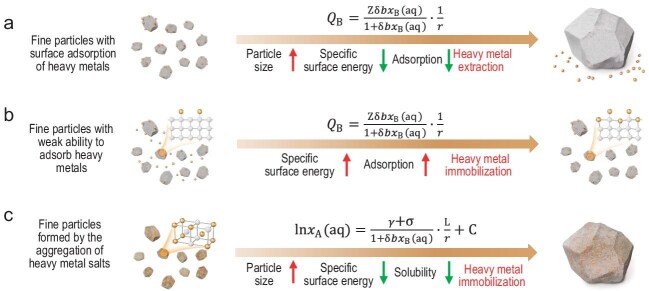
(a) Growth of fine particles reduces interfacial activity, promoting the desorption and extraction of heavy metals. (b) Defect creation enhances the adsorption and immobilization of heavy metals by fine particles. (c) The growth of fine particles formed by the aggregation of heavy metal salts reduces solubility and promotes heavy metal immobilization. Adapted from Ref. [6].

Furthermore, the proposed theoretical framework offers precise design guidelines for technologies aimed at controlled separation and stabilization of heavy metal pollutants. For example, in the detoxification and separation of adsorption-type fine particle wastes, optimizing particle size distribution and interfacial microstructure can improve the separation efficiency of heavy metals. In stabilization treatment, for different types of heavy metal-containing waste residues, regulating the specific surface energy (e.g. by introducing defects) of fine particles or controlling particle size through crystal growth can enhance the stability of heavy metal immobilization. This interfacial behavior-based technical pathway holds great promise and potential for providing long-term solutions for heavy metal pollution control (Fig. 1).

In summary, the multi-factor coupling theoretical framework for interfacial activity proposed in the work of Lin *et al.* overcomes the limitations of classical models and provides a quantitative and analytical tool for studying the interfacial behavior and processes of environmental fine particles. Their work not only deepens the understanding of the migration and transformation mechanisms of pollutants but also offers theoretical support for optimizing and advancing pollution control technologies. With the continuous progress of research in environmental interfacial chemistry, a deeper and more precise understanding of the regulatory mechanisms of interfacial behavior of fine particles will become an indispensable factor in environmental science and engineering research, providing essential scientific support for achieving accurate and sustainable pollution control and management.
